# Facebook Powered Measurement and Evaluation for Iron Folic Acid Health Intervention in India

**DOI:** 10.12688/gatesopenres.13047.2

**Published:** 2019-12-11

**Authors:** Drew Bernard, Chris McCullough, Sarah Francis, Avery Holton, Nadia Diamond-Smith

**Affiliations:** 1 Upswell, LLC, Seattle, WA, 98136, USA; 2 Communication, University of Utah, Salt Lake City, UT, 84112, USA; 3 School of Medicine, University of California San Francisco Medical Center, San Francisco, CA, 94143, USA

**Keywords:** Measurement and Evaluation, Facebook, Social Media, SBCC, Social Behavior Change Communication, M&E

## Abstract

As the media landscape changes and billions of people around the world turn to Facebook, Instagram, WhatsApp, and other social media platforms for information and social interactions, the need to develop effective methods of leveraging social media for social behavior change communication (SBCC) becomes increasingly important. Yet, in order for the public health sector to embrace social media for SBCC, we must have methods for measuring the impact of social media-based SBCC.

In this letter, we share a new approach for measurement and evaluation of social media-based SBCC campaigns. The approach was developed as part of an iron-folic acid health intervention targeting young women in two states in northern India; Uttar Pradesh and Maydar Pradesh.

## Introduction

This letter reflects insights learned during the first phase of a project with Upswell, Facebook, Ashoka University, and the Clinton Health Access Initiative titled ‘Leveraging Social Media Platforms For Social And Behavior Change Communications In India’. The project is designed to identify, develop, and measure the efficacy of strategies for leveraging Facebook for social and behavior change communication (SBCC) in India.

The overall project is made up of three distinct phases/campaigns three sequential SBCC campaigns in India - with each campaign (phase) informing the next. Each of the phases/campaigns will aim to increase awareness of and demand for iron and folic acid supplements among young women in the Indian states of Madhya Pradesh and Uttar Pradesh. At the same time, each of the three phases/campaigns will provide Upswell, The Bill and Melinda Gates Foundation, and grantees with learnings that will help improve the subsequent and future phases/campaigns. What we learn during phase one will make phase two more efficient and effective and what we learn during phase two will improve the outcomes of phase three. Finally, what we learn from the project will be shared with the public health sector, so others can use what we have learned to inform and improve their use of Facebook for SBCC.

We knew that a strong measurement and evaluation component would be vital to measuring the success of our SBCC campaign but found that there was not an established protocol or toolset for evaluating the real-world impact of social media campaigns. Traditional survey methodologies would not be a good fit; they would be slow and expensive, and it would be difficult to find and survey our target audience—active Facebook users—through any other medium besides Facebook.

Our goal was to evaluate knowledge and attitudes around anemia and iron-folic acid supplements, as well as iron-folic acid use, throughout our campaign. The method had to be reliable, statistically valid, repeatable, anonymous, and cost-effective. Ideally, we could also use the survey to gather data that would inform the SBCC campaign messaging and targeting.

This letter summarizes our first effort at tackling this challenge and what we have learned about the approach so far, and what will come next. The purpose is to discuss the methodology for using a combination of an online survey and Facebook for measurement and evaluation. At the time of writing, we are not prepared to present the findings from the effort, however, we will be publishing a peer-reviewed paper where that presents the findings in the near future.

## Methods

### Target audience

Our target audience, for both the campaign and the survey, was women who were active Facebook users, aged 15 to 49 and living in the states of Madhya Pradesh and Uttar Pradesh. According to data pulled from Facebook’s Audience Insights tool, there are 6.5 million women fitting that profile in Uttar Pradesh and 2.9 million in Madhya Pradesh.

Our goal for the survey was to gather enough responses on each survey to achieve a margin of error of approximately 3%.

Table 1 shows the population of our target audience that was on Facebook at the time of the survey (provided by Facebook’s audience targeting tool), the number of survey completions by targeting method (Reach targeting and Conversion targeting as tracked in Facebook Analytics), and the resulting margin of error. Data is shown for both Madhya Pradesh and Uttar Pradesh.

**Table 1.** Margin of Error calculation: = (SQRT((0.25/Survey Completions))*1.96)*(SQRT((Population on Facebook-Survey Completions)/(Population on Facebook-1))).

Survey Type	Audience	Population on Facebook	Completions	Margin of Error
Reach	Women ages 18-49 on Facebook In Madhya Pradesh as of 03/15/19	2,900,000	608	3.97%
Conversion	Women ages 18-49 on Facebook In Madhya Pradesh as of 03/15/19	2,900,000	1350	2.67%
Reach	Women ages 18-49 on Facebook In Uttar Pradesh as of 03/15/19	6,500,000	1346	2.67%
Conversion	Women ages 18-49 on Facebook In Uttar Pradesh as of 03/15/19	6,500,000	1539	2.50%

### The survey

The survey questions were developed in collaboration with UCSF and the University of Utah. The survey was initially developed in English and translated into Hindi with the help of Gates India and our partners at Rabbithole, an India-based marketing firm.

Once the questions were finalized, we created the surveys in Typeform, a cloud-based data collection app. We chose Typeform primarily because their forms are very well designed for mobile devices, which we knew would account for the vast majority of responses. Typeform also has Facebook Pixel and Google Analytics integration, which allowed us to gather some data about how users interacted with the form.

To separate responses by state and by targeting method (described below), we used four separate Typeform surveys. The surveys were identical, with the exception of one question about which district the respondent lived in which was state-specific Ad Targeting.

We distributed the links to our survey using ads created and delivered through Facebook’s Ad Manager. Ad Manager enabled us to target ads based on location, gender, age (among a huge variety of other variables).

On Facebook, a “campaign” can be any number of ads delivered to any number of target audiences with a specifically defined goal. Facebook then targets the distribution of your ads in a manner intended to maximize the value of your campaign. For example, if your campaign goal is “conversions,” Facebook will deliver your ads to users it believes are likely to “convert” -- in this case, click through the ad and submit a survey response. A “reach” goal, on the other hand, is optimized to display the ad to as many people as possible within your target audience.

Since Facebook’s ads algorithm targets conversion ads to people it believes are likely to convert (in our case clicking through and completing a survey), we were concerned that the audience sample would not be representative of the broader population. For this reason, we chose to test both “conversions” and “reach” in each state, for a total of four ad campaigns.

We also divided our overall audience into age group segments. Based on previous experience with other audiences around the world, we suspected that older users would be more likely to click through and complete the survey, and we wanted to test that theory. We also wanted to see if age played a role in helping to predict (or at least associated with) any of the variables. Additionally, if some age groups were underrepresented in the results, we could adjust our ad targeting to ensure we collected a meaningful sample.

Note that Facebook is not passing any information about individual users to our survey page. In fact, users did not share any personal information with us directly or through Facebook, and we cannot connect an individual user with their submission. Additionally, because we ran the survey using a third-party tool, Facebook does not see the data that users submit through the survey, and individual submissions are anonymous.

However, using Facebook’s analytics tool, which requires the implementation of a “Facebook Pixel” into the survey itself, we are able to see aggregated data about the users who clicked on our ads and submitted each survey. That allows us to make observations about our overall sample -- including age, gender, education, and location. This data should be helpful in testing the validity of our audience sample.

### Ad creative

The ads themselves were created by India-based creative agency Rabbithole with UpSwell guidance. Per our usual content strategy, we set out from the start to test a variety of messages that might motivate users to complete the survey while not biasing our sample by being specific about the topic or goals. The initial creative was informed by strategies that we have developed over the years. This strategy puts a heavy emphasis on the things that motivate people to take actions on social media. In this case, the intended action was to complete the survey which is a relatively high bar. We know from past experience that people are motivated to take actions that help them define themselves on social media. We also know that people are more likely to take action when they believe doing so will help other people. A review of the top performing content, below, provides a good example of how these concepts manifested in the creative.

### Retargeting and lookalike audiences

In addition to providing a way to test the validity of our audience sample, Facebook’s Pixel also provides a way to create “custom audiences” of people who have answered some or all of the questions in the survey. Facebook does not allow us to see which individuals have been added to a custom audience, but once a custom audience has enough users, Facebook allows us to target ads to the people in the audience. Additionally, custom audiences can be used to seed the development of, what Facebook calls, “Lookalike Audiences.” Lookalike Audiences use a large number of variables to identify people who are similar to the individuals who make up the initial custom audience. Although we have used Lookalike Audiences to powerful effect for other initiatives, we will need to test their effectiveness for SBCC campaigns at a future date.

## Response

We ran each campaign until the number of completed surveys resulted in a margin of error under 3% at a 95% confidence interval. We calculated the margin of error based on the size of the entire audience of female Facebook users in the states we were targeting, as found in Facebook Audience Insights ( Table 1). In total, we collected 4843 surveys, over a total of three days running the campaign.

Some highlights of the survey implementation process:

- Of the users who clicked through to the survey, 17.22% completed it. It took an average of 4 minutes and 44 seconds to do so.
- We spent a total of $4595.80 on Facebook advertising, for an average of $0.95 per response.
- There was a large difference in cost based on the campaign goal. The conversion campaigns averaged less than $0.30/completed survey, while the reach campaigns averaged $1.74/completed survey -- almost six times more expensive.
- Comparing our samples to the overall target population, we found that young women were underrepresented across the board (in terms of who clicked on the survey), and married women were overrepresented. That skew was more dramatic in the conversion campaigns, suggesting that the Facebook algorithms agree—older women are more likely to click through and complete the survey than younger women.

We also tested the ads by running a wide range of creative variants (see Table 2 and Table 3) and then focusing the advertising spend on the ads with the highest performing creative. And found that the “Recognize the heroine within” ad was the clear winner among every age group in both states. It performed almost twice as well as the runner-up (the anime “lying in the grass” ad).

**Table 2.** Top performing campaign creative.

Recognize the Heroine		Anime - laying in the grass		PopArt - Do you know your own power?	
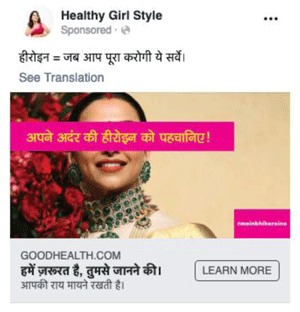		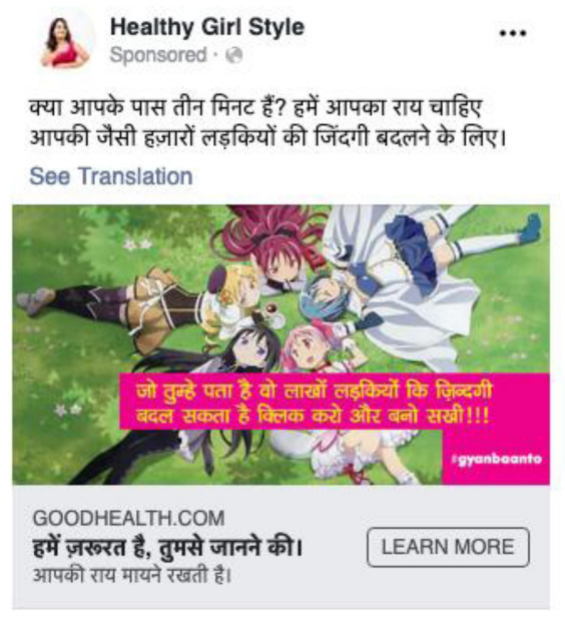		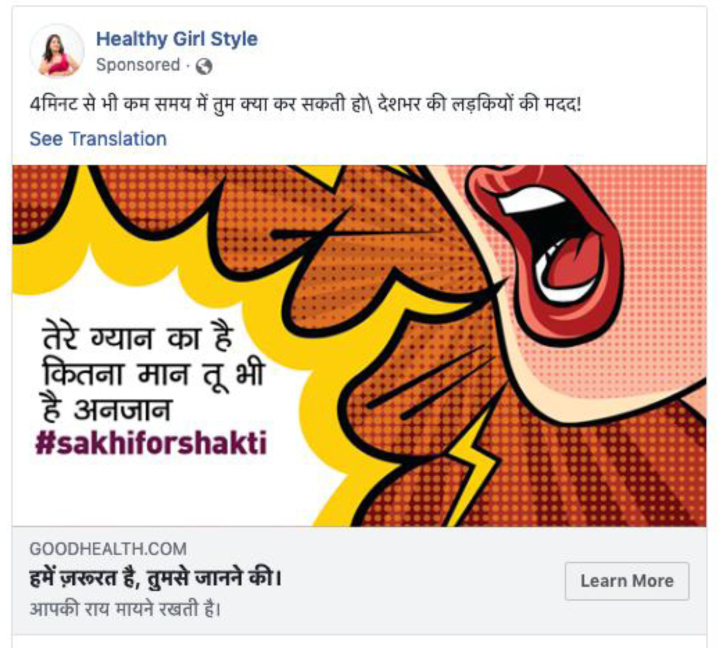	
**Post Text**	Heroine = YOU when you complete this survey	**Post Text**	Got three minutes? Quick we need your input to help thousands like you!	**Post Text**	What can you do in less than 4 Minutes? Help girls across India!
**Image** **copy**	Recognize the heroine within	**Image** **copy**	What you know can change millions of lives. Click to join your friends.	**Image** **copy**	You yourself are unaware of the true value of your knowledge
**Under the** **URL:**	We want to hear your thoughts Your input matters	**Under the** **URL:**	We want to hear your thoughts. Your input matters	**Under the** **URL:**	We want to hear your thoughts

**Table 3.** Top performing campaign creative (continued).

Anime - change the world		Just Girls	
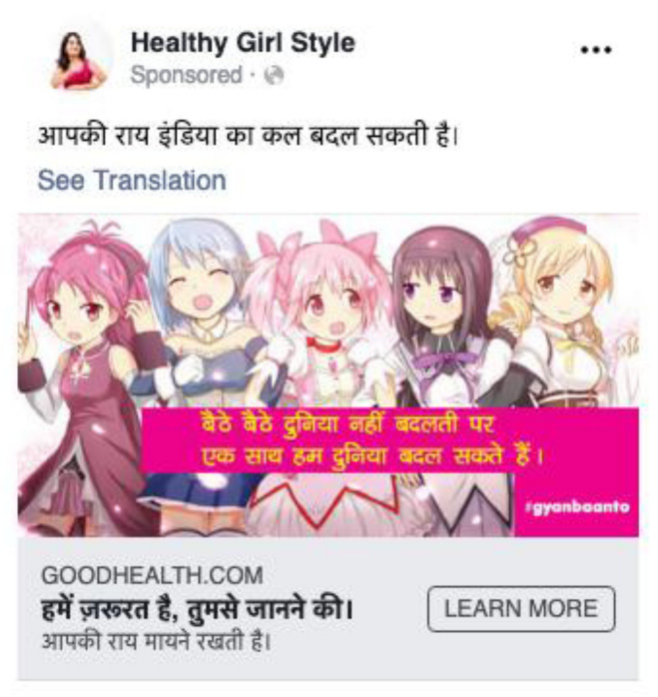		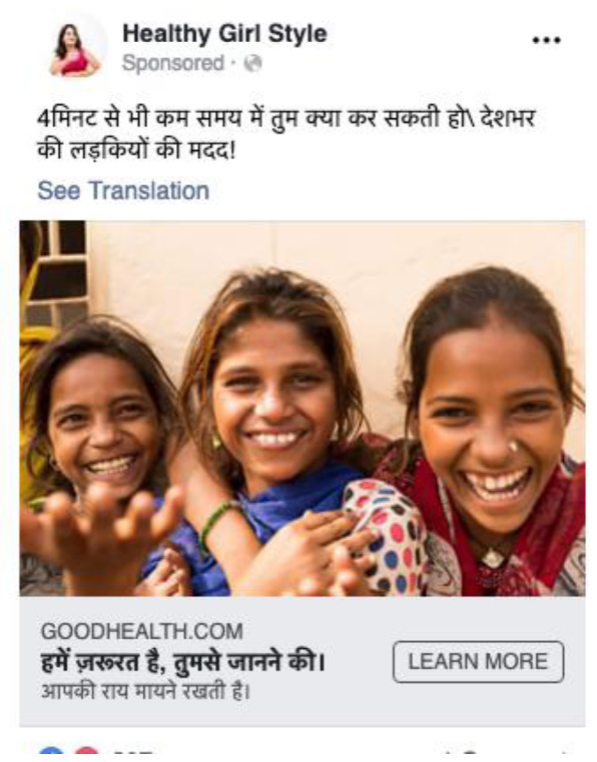	
**Post Text**	You have the power to help millions of women!	**Post Text**	What can you do in less than 4 Minutes? Help girls across India!
**Image copy**	The world doesn’t change if we keep sitting, we can only change it if we work together.	**Image copy**	None
**Under the** **URL:**	We want to hear your thoughts. Your input matters	**Under the** **URL:**	We want to hear your thoughts. Your input matters

## Questions and challenges

This is our first attempt at collecting survey data through Facebook. We are excited about the initial results, and they have raised a lot of questions we would like to explore more in depth.

Our biggest questions are about the validity of the audience sample itself. How representative are the 6.5 million women in Uttar Pradesh and 2.9 million women in Madhya Pradesh using Facebook of the overall population of women in those two states? Besides age and gender, what data can we use to measure how representative our sample is, and can we use that data to weigh our results? What can we do with the targeting, ad creative, and survey design to improve our sample? One approach to validate the audience would be to administer the same survey via traditional methods that do not rely upon Facebook for distribution and compare the results. 

We also found the process of running the survey using off-the-shelf tools to be very labor-intensive and highly error-prone. While using off-the-shelf tools with some custom coding is allowing us to test and learn, it is clear that we will need to find or build a solution that is more tailored to this kind of measurement and evaluation effort.

## Next steps

Over the coming months, we will be running Facebook advertising campaigns targeted at women under 40 in Uttar Pradesh and Madhya Pradesh designed to increase awareness of anemia in India, increase use of iron folic acid tablets and inform people about how to access anemia prevention resources.

To evaluate the impact of the campaign, we will re-run our survey during and post advertising campaigns to learn if we have shifted any attitudes.

## Data availability

No data are associated with this article.

