# Supplementary material for: Facebook Powered Measurement and Evaluation for Iron Folic Acid Health Intervention in India
Source: Gates Open Res. 2019 Dec 11;3:1536. Originally published 2019 Sep 12. [Version 2] doi: 10.12688/gatesopenres.13047.2 (PMC6880185; doi:10.12688/gatesopenres.13047.2)
Supplement: Peer review history

# Peer review: Reviewer response for version 1

**DOI:** 10.21956/gatesopenres.14169.r28116

**Published:** 2019-11-22

**Authors:** Lauren B. Frank

**Affiliations:** 1 Department of Communication, Portland State University, Portland, OR, USA

This Open Letter addresses an important challenge in the field of social and behavioral change communication – how best to measure campaign effectiveness on social media. You detail a process using Facebook tools to reach a specific audience of Facebook users, and your method allowed you to compare different choices for using Facebook to “reach” or “convert” users. Based on your findings, selecting the Facebook algorithm that promotes “conversion” yields less expensive cost per survey completion but a sample that is less representative of the population. These findings yield important information both for researchers and for campaign designers. I have a few recommendations for additions that I believe will add to the value of your letter.

1. In the Methods section, you specify that the goal sample size would give you a margin of error of approximately 3%. Can you provide the sample size and power calculations that yield that margin of error?
2. Please clarify whether you targeted or obtained any other variables in addition to location, gender, and age through Facebook’s Ad Manager.
3. In the Response section, you state that 17% of users who clicked through to the survey completed it. Does the process you used allow you to see how many Facebook users viewed survey links, how many clicked through, and how many completed it? A table showing how far users got in the process would be quite helpful for others planning similar recruitment efforts.

I confirm that I have read this submission and believe that I have an appropriate level of expertise to confirm that it is of an acceptable scientific standard.

# Response

**Published:** 2019-11-26

**Authors:** Drew Bernard

**Affiliations:** Upswell, USA

Thanks so much for your review and your suggestions. We are working to incorporate all of the items you suggest and hope to have an updated version submitted no later than December 6. 

 Thanks again. 

 Drew

# Peer review: Reviewer response for version 1

**DOI:** 10.21956/gatesopenres.14169.r27833

**Published:** 2019-10-28

**Authors:** Yu-Chao Huang

**Affiliations:** 1 Department of Indigenous Languages and Communication, National Dong Hwa University, Hualien, Taiwan

This paper is well written. I only have a few comments as follows:

1. First, on page 3, the authors described the process in which how the ads used in this paper were created; however, the detailed information such as the criteria of the judgment is missing. It would be helpful for readers to understand where the message strategies were taken from and how/why it could work for the target audience in this paper by providing more background information. In particular, as the paper illustrated the layouts of ads in table 2 and 3, readers would be curious about how these ads were initiated (by what theories or supporting evidence).
2. Second, the goal of this paper is to evaluate the Facebook powered measurement for social behavior change communication; therefore, the results of this paper are expected to give more information for readers to know about the effectiveness of using this method compared to others. For example, on page 3, the response section only showed their own outcomes such as the complete rate as 17.22% and the cost per response, without the counterpart results from the other methods such as the traditional online survey. Hence, the readers could not evaluate the effectiveness of their outcomes directly.
3. Last, the sample representativeness was mentioned as a major challenge for this paper. As a researcher who is interested in such a new method, I would expect to know further how it could be tested and validated even though it is understandable that the solutions could be in a follow-up study by the authors.

I confirm that I have read this submission and believe that I have an appropriate level of expertise to confirm that it is of an acceptable scientific standard.

# Response

**Published:** 2019-10-28

**Authors:** Drew Bernard

**Affiliations:** Upswell, USA

Yu-Chao - Thanks so much. We agree completely with your suggestions and will work to roll them into the paper after we receive one or two other reviews. Thanks again.
